# Network segregation and the propagation of misinformation

**DOI:** 10.1038/s41598-022-26913-5

**Published:** 2023-01-17

**Authors:** Jonas Stein, Marc Keuschnigg, Arnout van de Rijt

**Affiliations:** 1 Faculty of Behavioural and Social Sciences, University of Groningen, Groningen , The Netherlands; 2 Institute of Sociology, Leipzig University, Leipzig, Germany; 3 The Institute for Analytical Sociology, Linköping University, Norrköping, Sweden; 4 Department of Political and Social Sciences, European University Institute, Fiesole , Italy; 5 Department of Sociology, Utrecht University, Utrecht, The Netherlands

**Keywords:** Human behaviour, Complex networks

## Abstract

How does the ideological segregation of online networks impact the spread of misinformation? Past studies have found that homophily generally increases diffusion, suggesting that partisan news, whether true or false, will spread farther in ideologically segregated networks. We argue that network segregation disproportionately aids messages that are otherwise too implausible to diffuse, thus favoring false over true news. To test this argument, we seeded true and false informational messages in experimental networks in which subjects were either ideologically integrated or segregated, yielding 512 controlled propagation histories in 16 independent information systems. Experimental results reveal that the fraction of false information circulating was systematically greater in ideologically segregated networks. Agent-based models show robustness of this finding across different network topologies and sizes. We conclude that partisan sorting undermines the veracity of information circulating on the Internet by increasing exposure to content that would otherwise not manage to diffuse.

## Introduction

While only a modest fraction of news consumed online stems from platforms with a reputation for intentionally spreading lies^1,2^, the Internet is rife with misinformation that is less systematically generated and propagated by myriad individuals and organizations^3–6^. Political misinformation is particularly concentrated in partisan communities with ideologically aligned members, but partisan segregation is often not actually as extreme^7–10^ as suggested by such terms as “echo chambers” and “filter bubbles,” and varies across social media platforms.^11–14^. Here we ask whether the degree of ideological segregation of an online social network impacts the prevalence of misinformation spreading in it.

Previous studies of behavioral contagion have found that if an individual trait (e.g., ideology) associates with the sharing of whatever is to be diffused, then network segregation on that trait helps its propagation^15–17^. This happens because in segregated networks, information that diffuses meets susceptible individuals who pass it on to their susceptible neighbors^15,16^. By contrast, in integrated networks things that diffuse often meet individuals with low adoption propensity, and this ends their transmission. Our study is concerned with the spread of false versus true content. The present paper shows both theoretically and empirically that the diffusion-enhancing effect of network segregation is particularly strong for news that is otherwise too implausible to spread, while being nil for messages that also propagate in integrated networks. As a result, network segregation should increase the proportion of misinformation relative to true information circulating in an information system.

The exposure mechanism we propose operates as follows. Consistent with prior evidence we assume individuals are imperfect but better than random at telling truth from falsehood^1,18^ and do care about veracity^5,6^, but are more likely to share ideologically compatible messages^19,20^. We assume these dependencies to follow a linear probability model^21^. Accordingly, an individual’s susceptibility *s*—their propensity for sharing a message once they receive it—is the message’s baseline sharing probability *p*, which is positively related to veracity, plus ideological bias *b* for the ideologically aligned ($a=1$) and minus bias *b* for the misaligned ($a=-1$): $s = p + a \cdot b$. An individual’s probability of sharing a message equals their susceptibility *s*, where *p* and *a* are constrained such that *s* lies within the unit interval.

The positive spreading effect of network segregation found in earlier work is limited to messages that have low sharing probabilities (low *p*) and as a result fail to propagate in integrated networks. By contrast, co-location of ideologically aligned individuals who are more susceptible does not raise their exposure to high-probability messages (high *p*) in segregated networks, because these messages would reach them also in integrated networks. Because a message’s baseline sharing probability (*p*) is positively related to its veracity, the result is an increased fraction of false information circulating in a network.

**Figure 1:**
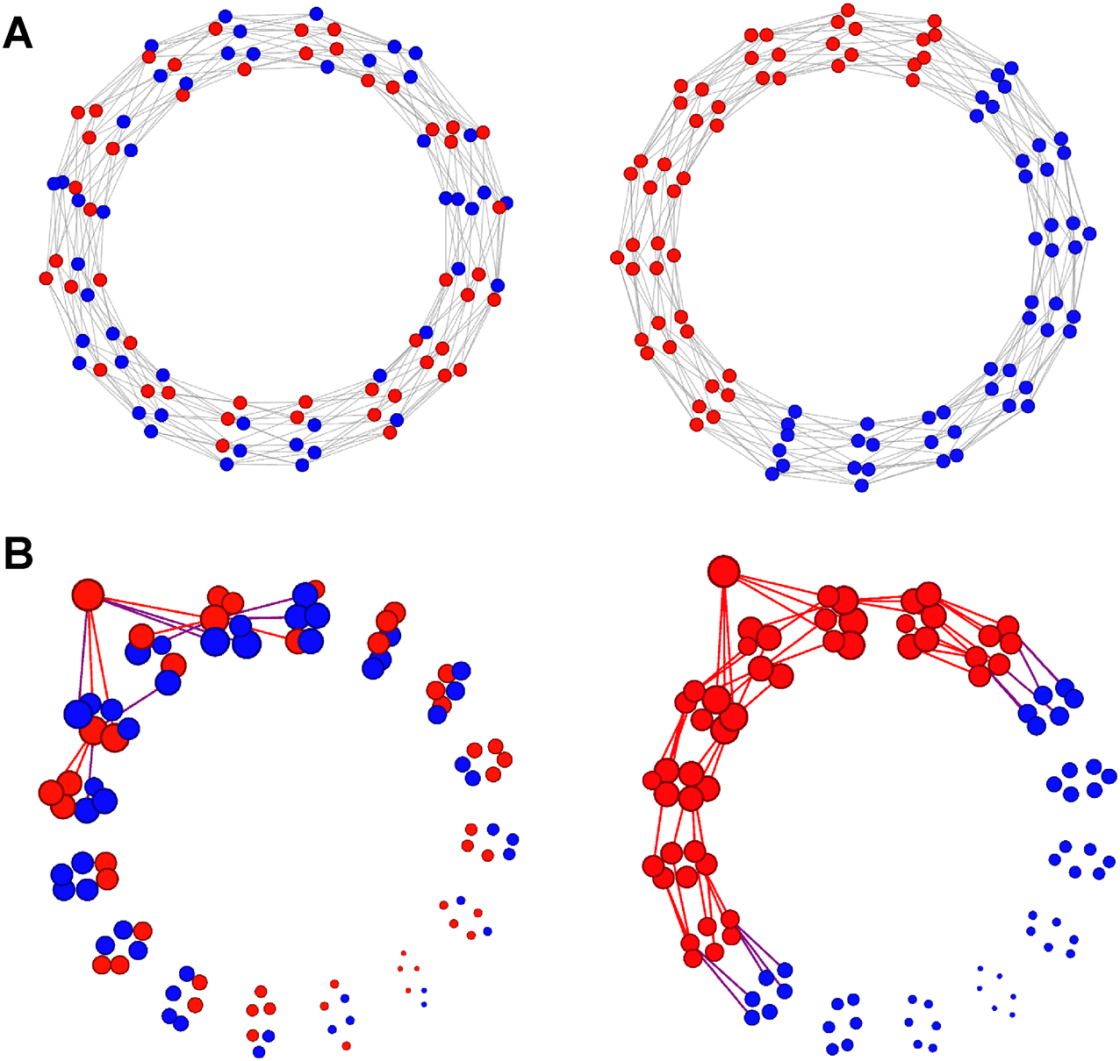
Experimental design. (**A**) Subjects were randomly assigned to either an ideologically integrated (left) or segregated network (right) in which red (blue) nodes represent self-identified conservative (liberal) participants (*N* = 96, degree = 6). (**B**) Each diffusion process (16 networks $\times$ 32 messages = 512 in total) was initiated by a random seed node, who received an ideologically aligned message, and had it automatically shared with their 6 neighbors. We show the observed propagation history of a false conservative-leaning message under ideological mixing (left) and homophily (right). Node size decreases with the shortest distance (1–8) to the seed node.

**Figure 2:**
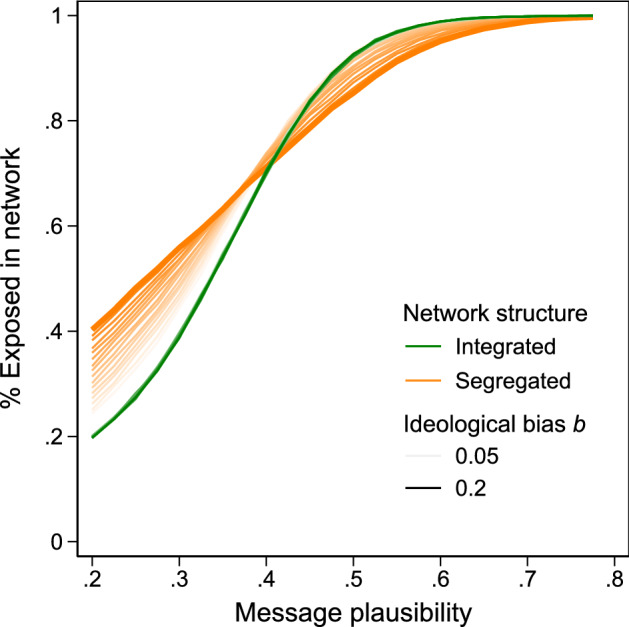
Theoretical expectations. We simulated diffusion processes in ring lattice networks as depicted in Fig. 1. Each diffusion process was seeded both in an integrated and in a segregated network (*N* = 96). We varied baseline sharing probability *p* (0.2–0.8) and ideological bias *b* (0.05–0.2), see Materials and methods. We measure exposure as the percentage of network nodes that have received a certain message. The model predicts that network segregation raises exposure to low-*p* content and decreases exposure to content with higher levels of *p*, and does so more for greater bias *b*. We report simulation results as connected lines across different *p*.

We illustrate the exposure mechanism using a stylized population, consisting in equal parts of conservatives and liberals (Fig. 1A). Consider a message with low baseline sharing probability *p* and conservative leaning. While some conservatives might share the message if it reached them, e.g., because their beliefs are biased or because they act in bad faith ($a=1$), no liberal would ($a=-1$, $p-b<0$). If this message enters an ideologically integrated population it cannot spread because conservatives live in neighborhoods in which too few will share the message for it to be able to keep propagating (Fig. 1B, left). Less susceptible nodes act as roadblocks to message diffusion and spreading dynamics die out as low-probability messages fail to reach those inclined to share it. By contrast, if the message enters an ideologically segregated neighborhood, it can diffuse because conservatives are surrounded by enough other conservatives that at least some neighbors will share it with them so that they, in turn, can pass it along (Fig. 1B, right). A segregated arrangement thus greatly exacerbates the spread of a message that only members of one camp would consider sharing. This prediction is shown in Fig. 2, which displays the results of 10,000 simulated diffusion processes in the segregated and integrated networks of Fig. 1 at variable levels of average sharing probability *p* and ideological bias *b* (see Materials and methods on the computational model): At low *p*, messages diffuse farther in segregated networks than in integrated networks. Integrated networks have greater capacity for weeding out false content and restrain the spread of misinformation—even if individuals’ behavior regarding what messages to share were equally biased in segregated as in integrated networks.

Now consider a conservative message with a higher baseline sharing probability *p*. Many conservatives would share it if they learned about it ($a=1$) and some liberals ($a=-\,1$) would share it as well ($p-b>0$). For example, some liberals retweeted messages reporting historically low approval ratings for Joe Biden. Conservatives are exposed to this plausible message regardless of network structure. Liberals, on the other hand, will be less exposed in a segregated arrangement because their cluster lacks conservative nodes with high susceptibility to pass on the message. Thus, for anything of moderate to high transmissibility, a segregated arrangement of resilient and susceptible individuals will limit exposure among those who are resilient to it^22^. This is so because transmission in the susceptible cluster is ensured, while spreading dies out in the resilient cluster for a lack in susceptible individuals to continue diffusion. This is visible in Fig. 2 showing that overall exposure to messages of moderate to high plausibility is somewhat lower in segregated networks. In conclusion, then, from the observation that low plausibility messages (which tend to be false) spread farther in segregated networks while messages of higher plausibility (which are more likely true) diffuse the same or even less so, it follows that segregation renders the fraction of misinformation circulating in the network higher.

The exposure mechanism we propose can be viewed as an application of the concept of herd immunity from epidemiology. The contrast between the spread of false versus true news is analogous to the difference between the spread of a low-$R_0$ versus a high-$R_0$ pathogen, where $R_0$ is the number of people an infected person can be expected to infect while contagious. The binary distinction between ideological alignment versus misalignment is analogous to a heterogeneous population of highly susceptible and mostly resilient individuals^23^, e.g., unvaccinated versus vaccinated individuals. The low-$R_0$ pathogen (false news) can only spread in segregated network clusters of unvaccinated (ideologically aligned) people^24^. In integrated networks, a sufficient fraction of vaccinated (ideologically misaligned) individuals provides herd immunity, preventing the pathogen (false news) from spreading. Thus, the size of the epidemic increases with the degree of network segregation^25^ and, in segregated populations, rates of resilient individuals need to be higher to reach herd immunity^26^. By contrast, the high-$R_0$ pathogen (true news) spreads both in segregated networks of unvaccinated (ideologically aligned) people as well as in integrated networks, where vaccinated (ideologically misaligned) individuals fail to provide herd immunity against spread of the highly contagious pathogen (true news). Only segregated network clusters of vaccinated (ideologically misaligned) individuals can withstand spread. Thus, for a high-$R_0$ pathogen segregation reduces rather than increases disease propagation.

A controlled test of the exposure mechanism requires, first, the ability to independently vary network architecture from *integrated* to *segregated*, holding all else constant—which is impossible in observational studies. Second, while previous studies only covered a single object of diffusion^15–17^ —and thus could not find variability in the segregation effect—we study 32 different objects of variable virality. Third, a number of studies have argued that in ideologically segregated environments individuals more readily share implausible messages that echo their political views^7,8,19,20,27–32^. To rule out the confounding of any such behavioral effects, individuals must be prevented from self-selecting into networks of homophilous friendships and remain uninformed about their neighbors’ ideological identities. We achieved these artificial conditions in a large social network experiment in which 512 diffusion processes spread in 16 populations (see Materials and methods and section S1 in the Supplementary Information for study details).

## Results

On the experimental platform, 16 empty networks with a predefined topology were each populated with 96 self-identified liberals and conservatives (Fig. 1A). Eight networks were maximally integrated and eight maximally segregated, representing two ideal-types that maximize our treatment as extreme cases of a continuum along which more and less segregated real-world online communities are positioned^7–9,19,20,32–34^. Each network constitutes an independent social system, allowing a macro-level test of the structural effect of network segregation on the spread of misinformation. We studied the propagation of 32 messages in each of the 16 networks, for a total of 512 propagation histories. Half of the 32 messages had a strong conservative (liberal) leaning, among which again half was backed up (“true”) or refuted (“false”) by academic research. Each participant was informed that messages that reached them could be true or false and asked to only share news they believed to be true. Figure 1B illustrates the exposure mechanism, showing the observed spreading of a false and implausible message (Table S1, row 13) in one of the integrated experimental networks and in one of the segregated experimental networks. In the integrated network, the message failed to reach many of its more likely adopters and died out quickly. In the segregated network, the most likely adopters were the first to be exposed, and the message diffused widely in the aligned part of the network, reaching individuals who were increasingly distant from the seed node and, ultimately, ideologically opposed individuals in the other segregated half.

**Figure 3:**
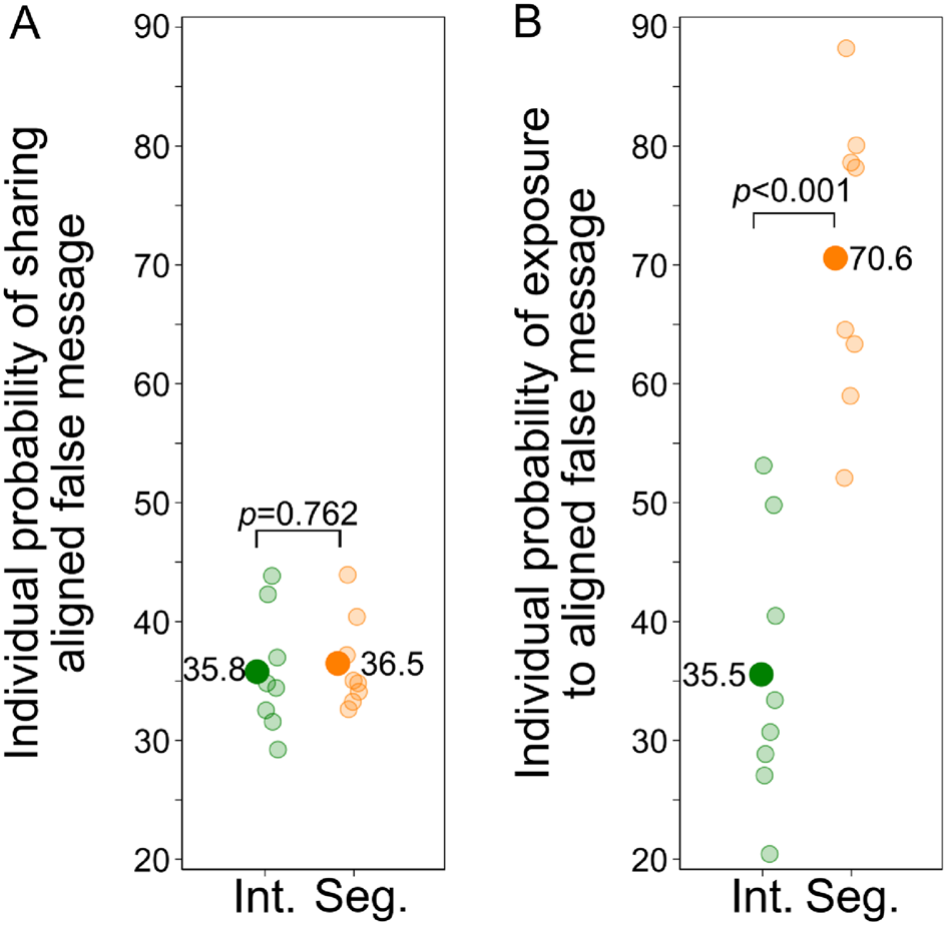
Individual sharing behavior and exposure to misinformation in integrated and segregated networks. (*A*) An individual who receives an aligned false message is just as likely to share it in an integrated network as in a segregated network (two-sided randomization test; *ATE*=0.7%, *p*=0.762, *N*=16). Each shaded circle represents one network. (*B*) By contrast, we find a sizable increase in the number of false aligned messages people receive in a segregated network, identifying the exposure mechanism that underlies the structural effect of network segregation on the spread of misinformation (one-sided randomization test: *ATE*=35.0%, *p*=0.0002, *N*=16).

### Individual sharing behavior vs. network exposure

Figure 3A demonstrates that individuals’ willingness to share false partisan messages did not increase in segregated (36.5%) vis-à-vis integrated networks (35.8%; two-sided randomization test: *ATE*=0.7%, *p* = 0.762, *N* = 16). This individual-level result indicates that our experimental design succeeded at precluding the behavioral effects of homophilious networks reported in prior literature^27–32^. Even though individuals’ responses to aligned false messages did not differ between network conditions, we find a marked increase of 35 percentage points in exposure to misinformation in segregated networks (Fig. 3B; 70.6% vs. 35.5%; one-sided randomization test: *ATE*=35.0%, *p*=0.0002, *N*=16). The sharing behavior among those who were particularly inclined to forward implausible ideologically connotated messages early in the diffusion process increased the exposure for subsequent recipients, allowing misinformation to diffuse. This finding lends strong support to the exposure mechanism driving the structural effect of network segregation (see section S2 for details on the statistical analysis). Figure S3 in the Supplementary Information extends this analysis to all 4 message types [aligned, misaligned $\times$ true, false] and corroborates the result: While sharing behavior is statistically indiscernible in segregated and integrated networks, subjects’ exposure to aligned misinformation increases in a segregated network architecture. Further, in section S4, we present additional results on behavioral differences between liberal and conservative participants.

**Figure 4:**
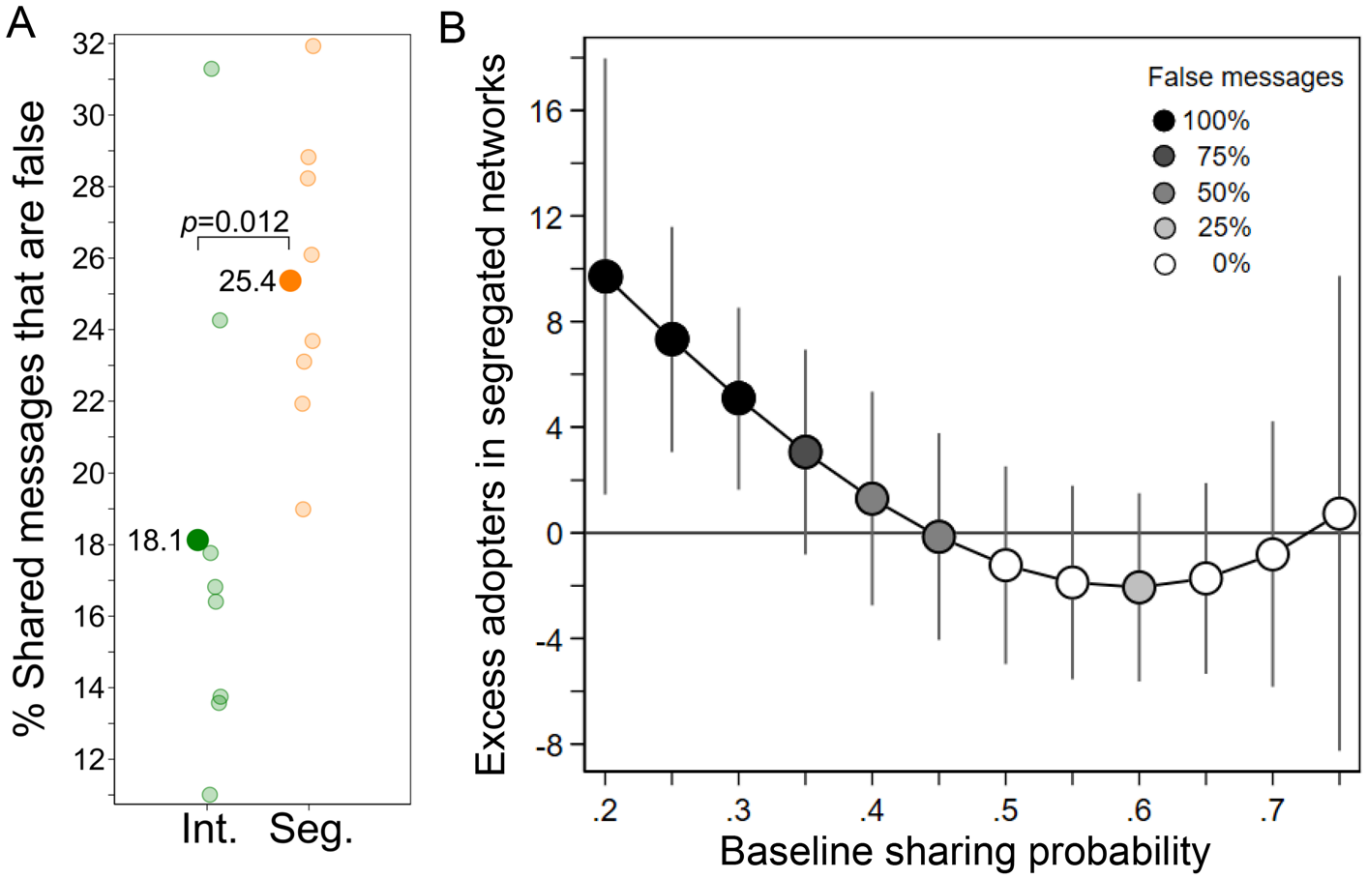
Misinformation proliferates in segregated networks. (*A*) Percentage of sharing decisions involving a false message in both network conditions. Each shaded circle represents one network. Consistent with our argument, we find that network segregation systematically increases the fraction of misinformation in the population (one-sided randomization test: *ATE* = 7.2%, *p* = 0.012, *N* = 16). (*B*) Difference in number of adopters between segregated and integrated networks by the messages’ baseline sharing probability (vertical lines denote corresponding 95% confidence intervals). The color shade indicates the fraction of false messages at different levels of sharing probability.

### Network segregation structurally favors misinformation

As a result of the exposure mechanism, segregated networks exhibited a greater prevalence of misinformation (Fig. 4A): The percentage of false messages as a total of all messages shared was 7.2 percentage points higher in segregated systems than in integrated ones. This average treatment effect is substantively large and statistically significant at the network level (one-sided randomization test: *ATE* = 7.2%, *p* = 0.012, *N* = 16).

Figure 4B differentiates the segregation effect by the messages’ baseline sharing probabilities, fitting a third-degree polynomial. We measure the baseline sharing probability as the fraction of subjects who shared a message in an independent experimental condition in which decision-making was monetarily incentivized to be truthful (see “Independent conditions” in section S1). Messages’ baseline sharing probabilities range from 0.22 to 0.77. The result illuminates how under network segregation false content increases as a fraction of all content: Network segregation clearly helps the propagation of low-probability messages, with the biggest boost given to the least plausible content. For high-probability messages, by contrast, the number of adopters in integrated and in segregated networks is statistically indiscernible. In conclusion, the proposed exposure effect is distinct from the generally positive spreading effect that network segregation is thought to exert on diffusion^15–17^.

Empirically calibrated simulations (see section S3) show that the segregation effect for low-probability messages generalizes to other commonly studied network topologies in which we vary network size, average degree, average path length, and the shape of the degree distribution. The network segregation effect on the prevalence of misinformation (Fig. 4A) arises in small world and scale free networks (Fig. S6) within a plausible range of network parameters where some, but not all information, has a chance to diffuse. The simulation results identify network topologies as particularly interesting, both theoretically and empirically, that support intermediate levels of virality. Topologies with properties where almost all information (very low average path length) or almost no information spreads (very high average path length) do not bring about segregation effects. Topologies with properties where some, but not all, information circulates are those that come closest to real-world networks and they are the ones where network segregation matters. Here, the spread of low-probability information increases once nodes are clustered in a segregated arrangement.

## Discussion

Our study demonstrates that ideological segregation will structurally increase the spread of false information across a social network: Network segregation creates local spreading infrastructures that bring together supply of and demand for ideologically aligned news that is believable to biased partisans but otherwise too implausible to propagate.

Stressing the role of network arrangements, our findings shift the focus of analysis and policy from micro-level behavior (e.g., campaigns targeting individuals to refrain from passing on misinformation^35,36^) to macro-level structure, highlighting the importance of ideological mixing of informational ecosystems as an instrument for mitigating the spread of “fake news.” While a social media platform’s act of exposing individuals to ideologically opposed alters by diversifying users’ networks could backfire at a psychological level—e.g., inciting further polarization of attitudes^10,37,38^—our result suggests that it would lower the prevalence of misinformation in the system overall. The experimental support for the exposure mechanism propagating aligned messages of low sharing probability in segregated networks suggests that social media platforms aiming to reduce misinformation should stimulate cross-partisan exchange and limit homophilous tie formation, for example by adjusting user recommendation algorithms so that roadblocks to implausible content can emerge.

Our network experiment achieved repeated realizations of the same result across multiple independent trials. Moreover, the result generalizes to other network topologies in agent-based simulation (see section S3) within a plausible range of network parameters. This lends strong support to the proposed exposure mechanism facilitating the propagation of misinformation in segregated networks.

Of course, our artificial experimental networks are a far cry from real-world platforms, in a number of respects. First, our networks are necessarily restricted in size due to natural bounds posed by synchronously participating experimental subject populations. As indicated in our simulations, segregation effects on the spread of misinformation can be expected to be disproportionately greater in larger networks, in which messages that go viral show starker contrast in absolute volume with messages that die out. Second, our design intentionally does not reveal neighbors’ ideological identities to participants, preventing effects of partisanship salience that could amplify the reported results. The politicized state of real-world platforms will likely render ideological bias stronger, further magnifying the structural effect of ideological homophily identified here. Third, social information such as counts of shares and likes may reinforce emergent differences in spread, further exacerbating segregated networks’ exposure effect on the spread of misinformation. With each of these amplifiers lacking, our study should be seen as conservative, suggesting that ideological sorting undermines the veracity of everyday news on the Internet to an even greater extent. Our analyses, further, center on cascade sizes, not on the speed of diffusion processes. It is likely that segregated networks speed up diffusion because a concentrated arrangement of adopters increases the number of possible diffusion paths and shortens the length of these paths compared to more integrated arrangements. Lastly, to provide a controlled experimental environment with manageable degrees of freedom, we did not consider diffusion dynamics in networks with different proportions of liberal and conservative subjects. Just like higher vaccination coverage can bring a population above the herd immunity threshold and prevent a disease outbreak, we would expect a piece of misinformation to proliferate less when lower proportions of individuals aligning with this information are present. Future research may investigate how such diffusion thresholds against misinformation are affected when different proportions of (mis-)aligned individuals are combined with varying levels of network segregation.

A crucial assumption in our study is that true news is on average more likely to be shared by the average user than false news is. This assumption circumscribes the validity of our main result, as without it we cannot deduce that network segregation increases the share of misinformation in a social network. However, the paper supports a much broader result that does not depend on this assumption and is applicable to anything that spreads between people: The direction of the network segregation effect on diffusion depends on the virality of the object to be diffused. Hard-to-diffuse objects are aided by segregation, while easy-to-diffuse things are hurt by it.

## Materials and methods

### Predictive model

We derived theoretical predictions of the network segregation effect from a simple model assuming two node types (“red” and “blue” agents). The model asks how network arrangement, combined with different susceptibilities across groups, affects the spreading of misinformation through the exposure mechanism. We placed 96 nodes on a ring lattice network with a degree of six. Networks were either segregated, in which nodes of the same ideology were maximally co-located, or integrated, where node ideology was allocated randomly (see Fig. 1). The diffusion of a message started after a seed node had shared the message with its six neighbors. In each round, all agents who had received the message in the previous round decided to share or discard it. A diffusion process stopped once no agent had received a message in the previous round (either because any agent shared it, discarded it, or never received it). An agent’s probability *s* to share a message depended on its baseline sharing probability *p* and the agent’s response to the message’s ideological bias *b*, such that $s = p + a \cdot b$, with $a=1$ for the ideologically aligned agents and $a=-\,1$ for the misaligned agents. First, we varied the baseline sharing probability from 0.2 to 0.8 (using increments of 0.025). Second, we varied ideological bias *b*, the degree to which nodes were more likely to share information that aligned with their ideology, and less likely to share information diverging from their ideology from 0.05 to 0.2 (using increments of 0.01). Red nodes thus shared a red message with *s* = baseline + ideological bias, and blue nodes shared a red message with *s* = baseline – bias, and vice versa. Hence, a message with baseline sharing probability of 0.6 and ideological bias set to 0.15 implied that aligned nodes shared the message with probability *s* = 0.75 and misaligned nodes with *s* = 0.45. Figure 2 displays the results of 10,000 simulated diffusion processes for each parameter combination (2 network types $\times$ 25 increments in baseline sharing probability $\times$ 16 increments in ideological bias), a total of 8 million simulation runs.

### Experimental conditions

We conducted the experiment on a dedicated online social network platform in which study participants received and shared news messages. 1536 US crowd workers were recruited through Amazon Mechanical Turk as experimental subjects. Data were collected during May 5–30, 2020. The study was approved by the ethics board of the European University Institute, Italy. All research was performed in accordance with relevant guidelines and regulations, and informed consent was obtained from all participants. We refer to section S1 in the Supplementary Information for study details, including subject recruitment and data quality. All experimental networks had the same undirected ring lattice structure^15,39^. We set the size of each network to 96 nodes—of which 48 nodes were reserved for liberal subjects and 48 nodes for conservative subjects—and each node was connected to 6 neighbors. Participants could only receive messages from and only share messages with their neighbors. Importantly, to isolate the structural effect of network segregation, neighbors’ ideology was not revealed. The ring lattice topology allows a high level of ideological sorting for a powerful test of the effect of network segregation on the spread of misinformation, as the local nature of a lattice permits a spatial partition of the graph into two highly clustered parts. In 8 *segregated networks*, ideologically aligned subjects were maximally co-located such that liberal participants populated one half of the lattice and conservatives the other half (fractions connected on both ends so that the ring structure persisted). Here, an average of 5.3 out of 6 network neighbors shared a participant’s ideology. In 8 *integrated networks*, participants were randomly distributed, and an average of 3.0 out of 6 neighbors shared a participant’s ideology. In both conditions, the ideological identity of participants was unknown to their neighbors, preventing behavioral effects of partisanship salience on news sharing. We thus expect individual sharing behavior to be the same, on average, in both network structures.

### Messages

In each network, we studied the propagation of 32 messages that fulfill three conditions: (1) they were original and hence unknown to participants, (2) a scientific measure of veracity was available for classification as “true” or “false,” and (3) they carried a political connotation that aligns with either a liberal or a conservative ideology. To allow timely evaluation, each message had a header indicating its topic and a body text of the length of a tweet ($\le$280 characters). Messages summarized the main research finding of a peer-reviewed social science article, which provided a ground truth. We selected articles that had not previously received widespread media attention, ensuring that messages were novel to subjects. The messages pertained to matters in politics, society, and science on which misinformation is commonly found in real-world online networks (Table S1). In half the cases, the finding supported a conservative viewpoint, and in the other half a liberal viewpoint. In half of the cases, we inverted or negated the message as to render it false. We ensured that the messages we introduced into the experimental networks met the scope conditions for our argument: the sharing of messages was positively correlated with their ideological alignment and false messages were deliberately constructed to be less plausible than true messages, allowing participants to distinguish true from false news. As an example, the false liberal-leaning message *“Cannabis use is not at all associated with any damage to adolescents’ cognitive functions, short or long term”* was shared less than the true conservative-leaning message *“Following Israel’s construction of a southern border wall between 2010 and 2013, annual numbers of illegal crossings declined”* and this difference was smaller for liberals. We calibrated the selection of messages in a pretest among a separate sample of 985 crowd workers. The pretest ensured that true messages were on average shared more than false messages and that message sharing was positively correlated with ideological alignment (see section S1 on “Message selection”).

### Seeding

We initiated each diffusion process by seeding 16 liberal and 16 conservative nodes (randomly selected without replacement) with one message each at the start of the experiment. The seed messages aligned with the node’s ideology and were automatically shared with the seed node’s 6 neighbors. The seeding of messages to ideologically compatible nodes explains part of the fact that *aligned* messages are shared more in segregated networks. It does not explain, however, our main result that aligned *false* messages are shared more in segregated networks.

### Subject experience

Each participant controlled a dashboard displaying the generic user names of their 6 neighbors and a message inbox (Fig. S1). A message appeared in their inbox once it had been shared by a neighbor (unless they had already received it from another neighbor). Participants were asked to “share” a message (with all 6 of their neighbors) if they believed it to be true, or “discard” otherwise. Subjects could not receive messages multiple times, could not revise their decisions, nor were they informed about the fraction of neighbors sharing a message. If subjects had emptied their inbox with no unread messages left after 8 minutes, their task was finished.

## Supplementary Information

Supplementary Information.

## Data Availability

The data and code that support the findings of this study are available for download at the Open Science Framework: https://osf.io/42qr8/.
