# Supplementary material for: Network segregation and the propagation of misinformation
Source: Sci Rep. 2023 Jan 17;13:917. doi: 10.1038/s41598-022-26913-5 (PMC9845210; doi:10.1038/s41598-022-26913-5)
Supplement: Supplementary file 1 — Supplementary Information. [file 41598_2022_26913_MOESM1_ESM.pdf]

Network Segregation and the Propagation of Misinformation

Supplementary Information

Jonas Stein, Marc Keuschnigg, and Arnout van de Rijt

Scientific Reports, 2023
https://doi.org/10.1038/s41598-022-26913-5

S1. Experimental design

Recruitment. We recruited a total of 1,992 participants from Amazon Mechanical Turk (MTurk). We limited participation to US residents and invited into each session an equal number of self-identified conservatives and liberals. US residency was validated by MTurk, which checks crowd worker location through the use of verified credit card addresses and social security numbers. In the US MTurk population, approximately 56% identify as politically liberal, 22% as moderate, and 22% as conservative[1]. We thus oversampled conservative crowd workers to achieve a balanced sample. First, we emailed US crowd workers who had previously participated in online research conducted by the ETH Zurich Decision Science Laboratory, our institutional partner for data collection. The laboratory's database contains more than 70,000 previous participants who gave informed consent to having their MTurk IDs stored and to being contacted by the lab. Emails were sent out in slices of several thousand, asking previous participants who identify as conservatives to take an online survey. The email contained a link to a MTurk Human Intelligence Task (HIT) that paid US$0.45 for a 3-minute survey. Second, we simultaneously posted the HIT on MTurk, visible to all US crowd workers. We limited participation to reliable crowd workers who reacted quickly to the posting of the HIT. Upon acceptance of the HIT, all potential participants—recruited either from the lab's database or directly from MTurk—answered a standard item for ideological self-identification[2]: "Here is a 7-point scale on which the political views that people might hold are arranged from extremely liberal to extremely conservative. Where would you place yourself on the scale? Extremely liberal (1), liberal (2), slightly liberal (3), moderate or middle of the road (4), slightly conservative (5), conservative (6), or extremely conservative (7)." We categorized participants into liberals (1–3) and conservatives (5–7), and we excluded moderates (4) from further registration and they received payment through an exit code. Liberals and conservatives were invited to sign up for a follow-up experiment, and they were able to subscribe to an experimental session scheduled within 6 hours after posting of the HIT. Anticipating no-shows, we allowed the number of sign-ups per ideological camp to be double the number of network nodes reserved for them per session, ensuring full occupancy of all available network positions. Upon completion of the registration phase, the crowd workers received payment and were assigned a MTurk qualification token making them eligible for participation in their scheduled session. The same token disabled multiple participation.

Subject experience. Before the experiment started, each participant received an overview

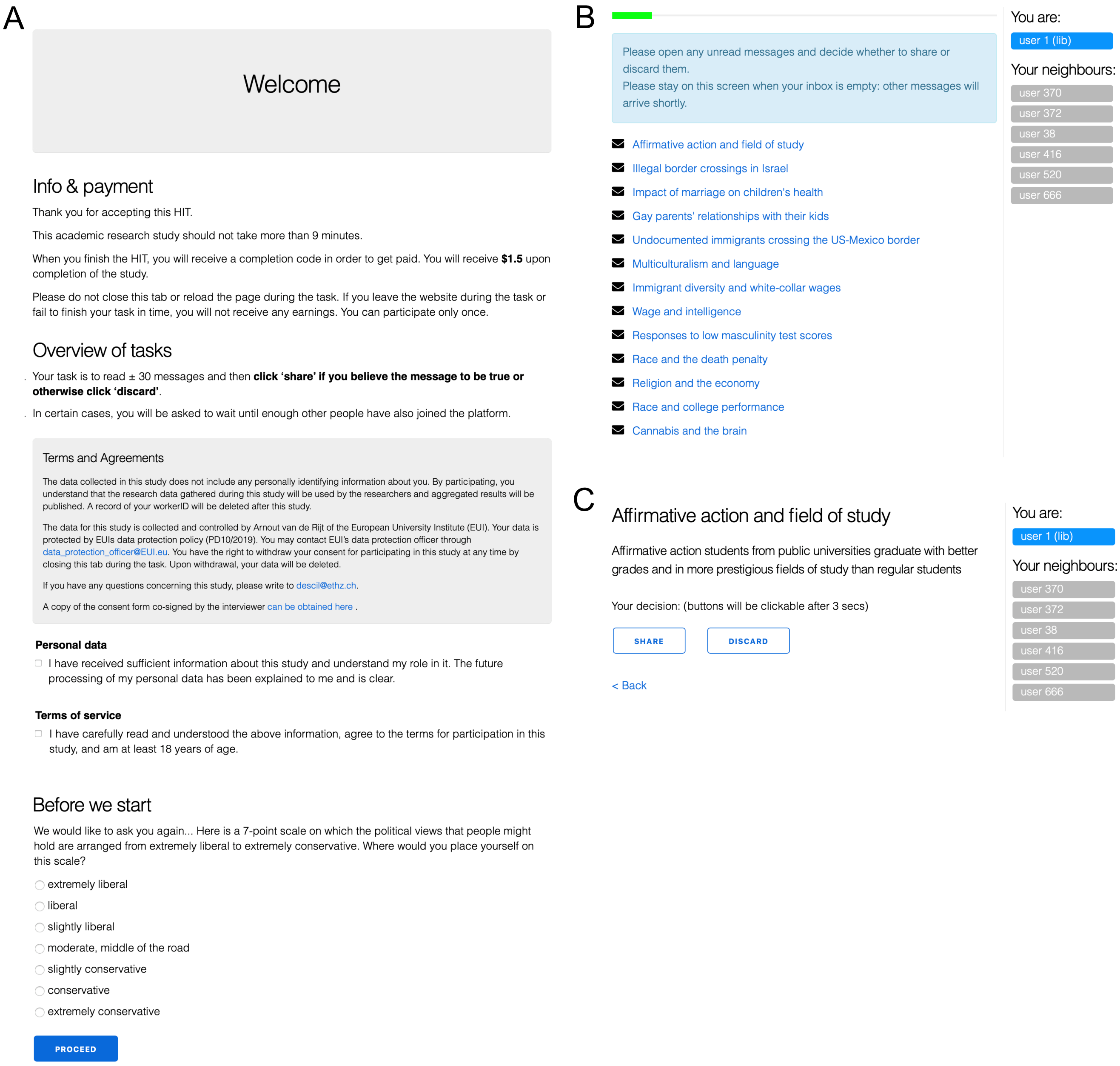

Fig. S1. Interface. (*A*) General instructions. (*B*) Dashboard showing message inbox, the 6 network neighbors, and an 8 minute time bar. (*C*) Decision screen showing example message text (no. 26) and the crucial 'share' and 'discard' buttons.

of tasks, was informed about the duration and payment of the study (approximately 9 minutes, US$1.5), and was asked to provide consent (see Fig. S1). Participant pay grosses up to US$10 per hour (6.67 $\times$ US$1.5), exceeding the US minimum wage. Each participant then controlled a dashboard displaying a message inbox and the generic user names of their network neighbors (Fig. S1). Importantly, to isolate the structural effect of network segregation, neighbors' ideology was not revealed. Once a message

had been shared by a neighbor, the message appeared in the participant's inbox. Each message had a short header indicating its topic and, if clicked, its content appeared in a pop-up window. Participants were asked to "share" the message with their neighbors if they believed it to be true and "discard" otherwise. Either decision removed the message from the participant's inbox. If a participant shared a message, it appeared in all 6 of their neighbors' inboxes. Subjects could not receive messages multiple times, they could not revise their decisions, nor were they informed about the fraction of neighbors sharing a message. We chose this setup as to not confound the structural effect by other network phenomena such as complex contagion[3] and effects of partisanship salience that could amplify the reported results. Over the course of 8 minutes, inboxes were constantly updated. After 8 minutes, participants with an empty inbox exited the experiment. Participants with remaining messages (2.1% of participants) were allowed an additional 3 minutes to take their decisions but inboxes no longer received any new messages shared by network neighbors. Our data analysis covers the first 8 minutes of the controlled propagation histories. We restricted the duration of the experiment to avoid loss in participant attention and increase statistical power. Keeping the experiment going until all inboxes are empty would have dramatically increased participation time, with most subjects idling for long stretches of time. The according increase in overall participant pay would then have reduced the affordable subject population size. Limiting participation to 8 minutes nonetheless allowed 97.9% of participants to empty their message inbox in time.

Message selection. To attain true messages unknown to participants, we sifted through recent issues of reputable general science and social science journals, from which we selected 72 empirical results that were sufficiently politically charged to be identified as consistent with either a liberal or conservative ideological leaning. We summarized each finding in a text of the length of a tweet. The general topics of the 72 messages were politics, society, and science. Half of the messages supported a liberal viewpoint, the other half a conservative one. We then created 72 false messages by inverting or negating each message, which left us with 72 true-false pairs, or 144 messages in total. We calibrated the selection of messages used in the experiment in a pretest among a sample of 497 liberal and 488 conservative crowd workers. Each participant received a random set of 24 (out of the 144) messages and was asked to "share" each message according to their perceived veracity, whereby each participant received either the true or the false version of a message only.

	Message	False	Lib.	p	b
1	Following Israel's construction of a southern border wall between 2010 and 2013, annual numbers of illegal crossings declined	0	0	0.65	0.09
2	Children born to married parents have slightly better health at age 5 than children born to cohabiting parents	0	0	0.60	0.10
3	On average, rich people are more likely to perform well on intelligence tests than poor people	0	0	0.54	0.07
4	Due to leading professionals and technological advantages, cancer patients in the US have better survival chances than in most European countries	0	0	0.52	0.18
5	Blacks and Hispanics admitted to elite US colleges perform more poorly than Asians	0	0	0.52	0.07
6	Sexual infidelity is more common among unmarried couples who live together than among married couples	0	0	0.44	0.17
7	Air pollution kills slightly more people than smoking does	0	0	0.42	0.13
8	In countries run by left-wing political parties, immigrants are more likely to be worse at speaking the official language of the new country	0	0	0.37	0.18
9	When more people attend church, the economy grows	1	0	0.33	0.18
10	Playing violent computer games makes gamers much more likely to be violent than before	1	0	0.32	0.11
11	US states with the death penalty have had much lower homicide rates throughout history as compared to US states without the death penalty	1	0	0.30	0.17
12	Foreigners burden the German federal budget, causing a negative balance	1	0	0.28	0.13
13	Children raised by homosexual parents experience more mental health issues than children raised by heterosexual parents	1	0	0.26	0.13
14	Scientists have observed that Arctic sea-ice loss has no association with human CO2 emissions	1	0	0.24	0.21
15	A vast majority of homosexual parents report worse relationships with their children than heterosexual parents	1	0	0.22	0.12
16	Police officers speak with equal respect to all community members, regardless of race, in stop-and-frisk encounters	1	0	0.22	0.17
17	White people are more likely to favor the death penalty than black people	0	1	0.77	0.13
18	When mens' masculinity is called into question, they are more likely to respond with a homophobic attitude	0	1	0.74	0.14
19	Human-induced CO2 levels in the air have resulted in increased chances of wildfires in California	0	1	0.69	0.21
20	Global warming is linked with negative effects on mental health	0	1	0.67	0.21
21	The greater the share of immigrants in a Western country, the more accepting native citizens are of new immigration	0	1	0.61	0.13
22	The greater the immigrant diversity in a city, the higher the wages for white-collar jobs	0	1	0.59	0.13
23	The number of undocumented migrants attempting to cross the US - Mexico border has steadily declined over the past two decades	0	1	0.55	0.13
24	Self-driving cars are better at detecting white pedestrians than black ones	0	1	0.39	0.06
25	Europe leads the ranking for the best ranked universities in the world	1	1	0.59	0.10
26	Affirmative action students from public universities graduate with better grades and in more prestigious fields of study than regular students	1	1	0.47	0.13
27	Children whose parents live together but are unmarried are less likely to experience their parents' separation than children whose parents are married	1	1	0.39	0.04
28	Businesses owned by women are much more successful than comparable businesses owned by men	1	1	0.38	0.11
29	Among white people in the US, unmarried couples who live together live significantly longer than their married counterparts	1	1	0.37	0.01
30	Men in same sex relationships are much more likely to be in relationships that are serious, monogamous, and sexually exclusive	1	1	0.37	0.10
31	Married people are much more likely to suffer from depression than single people	1	1	0.31	0.07
32	Cannabis use is not at all associated with any damage to adolescents' cognitive functions—short or long term	1	1	0.30	0.10

Table S1. Messages. The true messages we introduced in the experimental networks were on average shared more than the false messages. p refers to messages' baseline sharing probability as measured by their fraction of shares in an incentivized independent condition. Sharing was positively correlated with ideological alignment. b refers to messages' ideological bias as measured in a non-incentivized independent condition as the absolute difference in the sharing probability among liberal minus the sharing probability among conservative participants, divided by two.

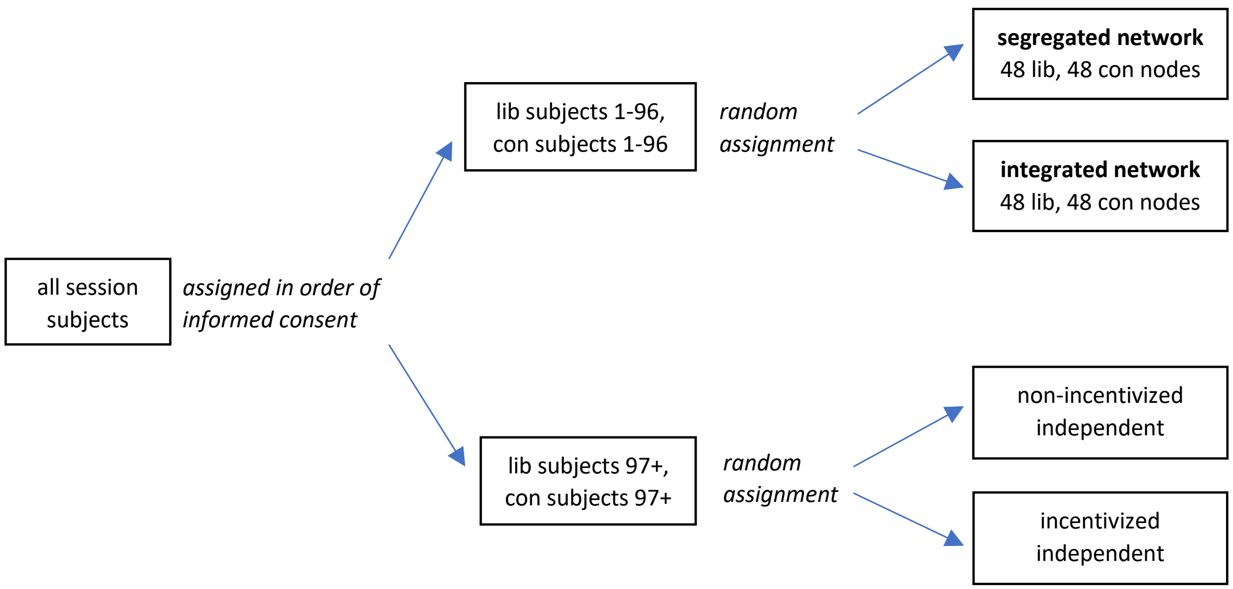

Fig. S2. Subject assignment. The first 96 liberal and first 96 conservative subjects giving consent were randomly assigned to an ideology-matching node in either the segregated or the integrated network condition. Excess subjects were randomly assigned to the non-incentivized or the incentivized independent condition.

The "sharing" behavior elicited in this pretest allowed us to rate messages according to their baseline sharing probability and ideological bias. We calculated a message's baseline sharing probability as the number of shares among both liberal and conservative participants. A message's ideological bias is defined as the absolute difference in the sharing probability among liberal participants minus the sharing probability among conservative participants, divided by two. For the experiment, we sought true (false) messages that scored high (low) on baseline sharing probability and carried a strong ideological connotation. We selected 32 news messages accordingly (Table S1)—16 true (8 conservative, 8 liberal) and 16 false (8 conservative, 8 liberal). The pretest ensured that true messages were on average shared more than false messages and that sharing was positively correlated with ideological alignment. We verified the properties of the 32 selected messages in independent conditions run in parallel to the experiment.

Subject assignment. We conducted 8 experimental sessions, each featuring one integrated and one segregated network with 96 nodes each. Upon arrival at their scheduled session, we assigned 96 liberal and 96 conservative participants (N=1,536) to a randomly chosen network and within that network to a randomly chosen node with matching ideology (see Fig. S2). Participants of either ideology were selected for assignment to the two networks by the order in which they gave informed consent. Excess participants arriving late (97th and later) were redirected to one of two independent conditions.

Independent conditions. For those sign-ups not randomized into network conditions (N=456) we varied the payoff structure in two independent conditions. We use both conditions to validate message calibration. In both conditions, each subject was asked to independently evaluate each of the 32 messages, with half of the participants receiving a flat-fee payment (US$1.50) upon completion, and the other half receiving a show-up fee (US$1.50) plus US$0.10 for each true message shared and for each false message discarded. Each true message discarded and each false message shared would reduce their payment by US$0.10. The independent conditions confirm that crowd workers reacted in predictable ways to the veracity and the ideological leaning of messages. First, participants, on average, could tell apart true from false news: Regardless of incentivization, baseline sharing probability was a reliable predictor of actual message veracity (point-biserial correlation r=0.73 [t=5.780, p<0.001] with incentives and r=0.63 [t=4.489, p<0.001] without). Incentivization significantly improved correct veracity assessment, with true messages shared somewhat more often in the incentivized than in the non-incentivized condition (56.4% vs. 52.0%, t=3.790, p<0.001) and false messages somewhat less often (32.5% vs. 35.4%, t=2.617, p=0.009). This reveals a tendency for ideological convenience to corrupt assessments of veracity, while at the same time corroborating the assumption that both with and without incentives, subjects were able to spot and discard false content. In order to crowd out subjects' ideological impulses when measuring messages' baseline sharing probability, we use for Fig. 4B the sharing rates elicited in the incentivized condition. Second, participants shared ideologically aligned messages significantly more often than misaligned ones. This was the case under incentives (aligned: 53.5% vs. misaligned: 35.3%, t=16.11, p<0.001) and particularly so under flat-fee payment (aligned: 55.8% vs. misaligned: 31.7%, t=21.13, p<0.001). We consider the non-incentivized elicitation a better measure of ideological bias because it lacks the monetary incentives that may crowd out ideological impulses. Table S1 lists each message's baseline sharing probability p (as measured in the incentivized condition) and ideological bias b (as measured in the non-incentivized condition).

Data quality. We took a number of steps to assure data quality. Subjects could not instantaneously participate but had to sign up for a given session and hours later arrive at the session on time. Late-shows were not allowed entry into experimental networks. Subjects who reported a different ideology during the recruitment HIT than before the actual experiment (i.e., changed camp) were not allowed into the experiment. Subjects who varied in the strength by which they identified as either liberal or conservative were allowed to participate. This was the case for 5.5%, or 84 of the 1,536 subjects in

the experiment. During the experiment, a three-second timer (see Fig. S1*C*) prevented subjects from making sharing decisions without having had an opportunity to read the message. Our independent conditions verified that subjects' sharing decisions favored ideologically aligned messages (see Table S1), both when incentivized to share true messages and when not. In the networked conditions, only 32 out of 1,536 subjects did not finish their tasks (drop out was thus 2.1% or, on average, 2 out of 96 participants) and only 6 subjects idled during the first five minutes of the experiment.

S2. Statistical analysis

Fig. 3A addresses our expectation that individuals were just as likely to share an aligned false message in a segregated network as in an integrated network. We calculated, for each network, individual sharing probabilities as the number of times subjects shared an aligning false message, divided by the total number of subjects exposed to an aligning false message.

Fig. 3B visualizes subjects' probability of being exposed to an aligned false message. We calculated individual probabilities of exposure as the number of times a subject received an aligned false message, divided by the total number of aligned subjects. Extending on Fig. 3, we show subjects' probability of sharing of and exposure to all 4 message types [aligned, misaligned × true, false] in Fig. S3 below.

Fig. 4A reports the test of our hypothesis that the share of misinformation is greater in segregated than in integrated networks. We calculated the percentage of false messages as the number of shares of false messages, divided by the total number of shares of true and false messages combined.

		Fig. 3A (two-sided)		Fig. 3B (one-sided)		Fig. 4A (one-sided)	
Test	N	Statistic	p-value	Statistic	p-value	Statistic	p-value
permutation test mean	16	2.6%	0.429	+22.8%	0.0004	+3.6%	0.034
two-sample t-test	16	$t = 0.9$	0.404	$t = 5.9$	0.0001	$t = 2.6$	0.010
rank-sum test	16	$z = 0.6$	0.553	$z = 3.3$	0.0003	$z = 2.2$	0.014
paired t-test	8	$t = 0.7$	0.497	$t = 6.2$	0.0002	$t = 2.9$	0.012
signed-rank test	8	$z = 0.6$	0.641	$z = 2.5$	0.012	$z = 2.1$	0.018

Table S2. Alternative tests for the results reported in Figs. 3A, 3B, and 4A.

For all three figures, we calculated y-axis values separately for each message in each network. To avoid undue influence of outliers, we aggregated across messages within each network using the median value, leaving us with one value per network. Aggregating

over messages is necessary because messages within a given network do not constitute independent observations. We conducted permutation tests of a zero average treatment effect, of which the results are reported in the main text. As Table S2 shows, results are similar when using different tests, among which a permutation test of the ATE with mean fractions per network, a two-sample t-test, and a rank-sum test. Result are also robust when making only within-trial comparisons, in paired t-tests and signed-rank tests.

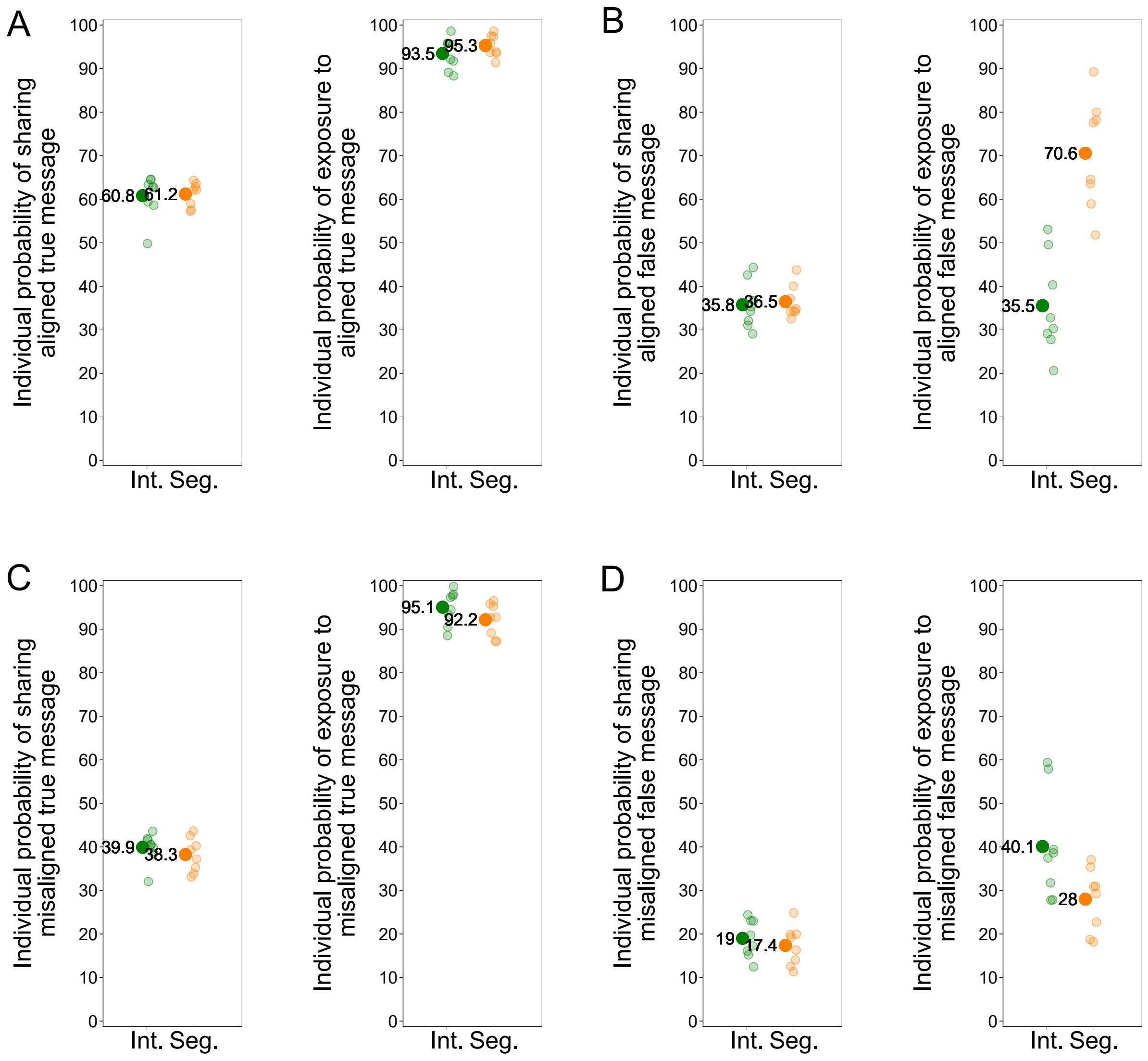

Fig. S3. Sharing of and exposure to messages in integrated and segregated networks. (A) Aligned true messages, (B) aligned false messages (cf., Fig. 3), (C) misaligned true messages, (D) misaligned false messages. Each shaded circle represents one network. Individual sharing probability equals the number of times subjects shared a respective message type, divided by the total number of aligned (or misaligned) subjects exposed to it. The individual probability of exposure equals the number of times a subject received a respective message type, divided by the total number of aligned (or misaligned) subjects.

Fig. 4B differentiates the segregation effect by messages' baseline sharing probability p. We estimated the predicted difference in adopters of false messages in segregated vis-à-vis integrated networks, y, by p using a polynomial parametrization of the ordinary least squares regression $y = \beta_0 + \beta_1 p + \beta_2 p^2 + \beta_3 p^3 + \epsilon$. The model minimizes messages' squared distances to a flexible best-fit function that relates baseline sharing probability to the network segregation effect. We report the estimated segregation effect (and 95% confidence intervals) for twelve 5-percentage point bins of p, ranging from 0.20 to 0.75.

S3. Empirically calibrated agent-based model

Building on the theoretical model that predicted that segregation structurally increases the prevalence of misinformation in networks (Fig. 2), we generate an empirically calibrated model in which agents' micro-behavioral parameters match the subjects' behaviors observed in the experiment. The calibration rests on observed parameters that capture how people react to message plausibility and the ideological leaning of messages. The calibrated model, first, allows us to verify if we can reproduce the macro-level results of the experiment. Second, the model permits exploring generalizations to different network topologies in which we vary size, average degree, average path length, and the shape of the degree distribution. The calibrated baseline simulation (ring lattice, degree=6, N=96) closely reproduces the quantitative targets elicited in the experiment (Fig. S5). The network segregation effect also arises in different network topologies (Fig. S6) within a plausible range of network parameters where some, but not all information, has a chance to diffuse.

Agents and messages. As in the predictive model, empty networks consisting of N nodes are populated by equal numbers of conservative and liberal agents. As in the experiment, agents receive and share with their network neighbors up to 32 messages with liberal or conservative connotation. Upon initialization of the simulation, each message is sent to a seed agent with aligning message ideology and, as in the experiment, the message is automatically shared with their neighbors. In each simulation round, all agents who received a message in the previous iteration decide to share or discard it. Agents' sharing decisions are probabilistic and, as in the predictive model, depend on the a message's baseline sharing probability p and the agent's response to the message's ideological bias b, such that an agent's probability of sharing a message equals $s = p + a \cdot b$, with $a = 1$ for ideologically aligned agents and $a = -1$ for misaligned agents, and except for below-zero and above-one susceptibilities which translate into $s = 1$ and $s = 0$, respectively.

		Message			
		Conservative		Liberal	
		True	False	True	False
Subject	Cons.	58.9	42.7	42.0	26.5
	Lib.	38.1	13.7	65.6	36.3

Table S3. Calibration of agents' sharing behavior. Summary statistics for calibrated message sharing probabilities. Means of percentages from the networked experiment. N(messages)=32.

Calibration. We calibrate the probabilities with which agents of a given ideology share ideologically aligned or misaligned true and false messages using each message's respective sharing rate elicited in the networked conditions of the experiment (Table S3). We define a message's sharing probability as the total number of experimental subjects who decided to share it divided by the total of all "share" and "discard" decisions. For each message, we aggregate sharing probabilities separately for liberal and conservative participants per session and we average, for each camp, over sessions. Across camps, sharing probabilities are higher for true messages (51.2±17.1) as compared to false ones (29.8±14.0; t=5.5, p<0.001), reflecting the higher plausibility of true messages. Sharing probabilities are also higher if ideologies of participants and messages align (50.9±16.6) as opposed to diverging ideologies (30.1±15.0, t=5.3, p<0.001), capturing how people with different ideologies react to the ideological leaning of messages.

Diffusion. Each iteration starts with those agents who have newly received a message to decide whether to share or discard it. A conservative (liberal) agent shares a message if a uniformly random number between zero and one is lower than the calibrated sharing probability of conservatives (liberals) for the respective message; and otherwise discards the message. When all agents have made their decisions, the iteration ends with the sending out of shared messages to their network neighbors. The simulation run ends once no agent has received a message in the previous round. This happens when all diffusion processes have come to their natural end, either because any agent shared it, discarded it, or never received it. In parallel to the experiment, we measure the extent to which misinformation proliferates in terms of the percentage of false message shares divided by the total of all messages shared, measured by the median spread of a false (true) message in each network.

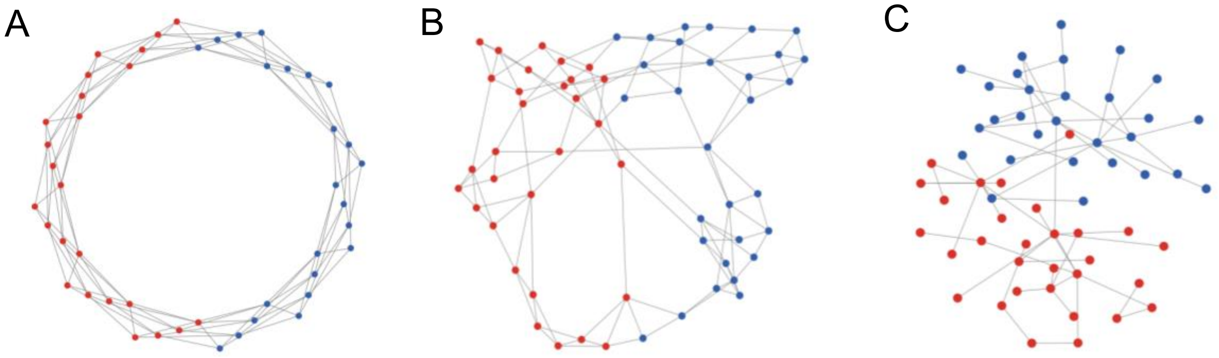

Fig. S4. Visualization of network topologies used in the simulation. (*A*) Hexagonal ring lattice. (*B*) Small world network. (*C*) Scale free network. For better visibility, here we show graphs of reduced size (N=48) and average degree (k=4).

Networks. All simulated networks feature an equal number of agents of either ideology, which we either arranged in a highly segregated pattern, with aligned agents maximally co-located, or randomly distributed across the network. In addition to the baseline simulation that considers an undirected hexagonal *ring lattice* as in the experiment, we simulate two commonly studied topologies, namely *small world* networks[4] and *scale free* networks[5], both of which share with real-world online networks the feature of short path length (Fig. S4). In the extrapolation models, we vary network size N and average degree k within each of the three topologies.

We construct small world networks by laying out nodes in a circle and letting them form links with k closest neighbors. We then randomly rewire each link in the network according to a default probability $r = 0.10$.

We build scale free networks using the Barabási-Albert algorithm where agents are added to the graph one at a time and form ties with m other agents according to $p_i = \frac{k_i^\alpha \cdot h_i}{\sum_j k_j^\alpha \cdot h_j}$. p_i is the probability of choosing agent i to connect with, k_i denotes the number of ties agent i already has, and k_j represents the number of ties of any other agent j. The exponent α influences the strength with which newly added agents preferentially connect to the most well-connected agents and thus determines the shape of the degree distribution. For $\alpha = 1$, which is the default value for simulations in which we vary size and average degree, a node's log(degree) is associated linearly to the log(frequency) of that node type. For $\alpha < 1$ this association is sublinear and for $\alpha > 1$ superlinear. h_i is a homophily parameter that conditions tie formation on agents' ideology. In segregated networks, tie formation is homophilic such that $h_i = 0.95$ when i and the newly added node are of identical ideology and $h_i = 0.05$ otherwise. To

preserve topological equivalence, integrated networks are constructed using the same parameters for h_i but node ideology is randomized once all nodes have been added and ties created. We vary average degree k in scale free networks by adjusting m, where $k \approx m \cdot 2$.

Throughout the different network typologies, we vary average degree k from 2 to 8, and consider networks the size of $N = 48$, 96, 192, and 384 (Fig. S6). We further report results in which we set $N = 96$ and $k = 6$ but vary $r \in \{0, 0.05, 0.1, 0.15, 0.2\}$ in small world networks and $\alpha \in \{0, 0.5, 1, 1.5, 2\}$ in scale free networks (Fig. S7). For each parameter combination, we report results for 1,000 simulation runs.

Results. First, the calibrated model reproduces the main result that network segregation structurally increases the prevalence of misinformation. The calibrated baseline (ring lattice, degree=6, N=96) matches the macro-level results elicited in the experiment (Fig. S5). Filled circles represent the percentage of false sharing decisions in integrated (green, 18.1%±6.6) and segregated (orange, 25.4%±4.2) experimental networks. Squares mark the mean simulation results for ring lattices of size 96 (16.1%±4.7 false sharing decisions in integrated and 26.4%±4.3 in segregated networks). Those are statistically indiscernible from the mean experimental results (integrated networks: t=1.224, p=0.222; segregated networks: t=0.676, p=0.499).

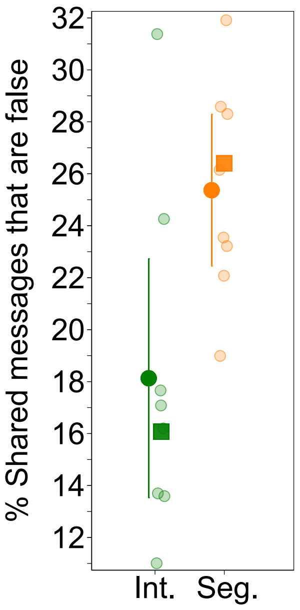

Fig. S5. Percentages of false sharing decisions in integrated and segregated simulated networks. The filled circles represent the calibration targets from the integrated (green) and segregated (orange) experimental networks (cf., Fig. 4A). Shaded circles represent each network condition's 8 experimental trials. Squares indicate mean results from the calibrated baseline model (ring lattice, degree=6, N=96).

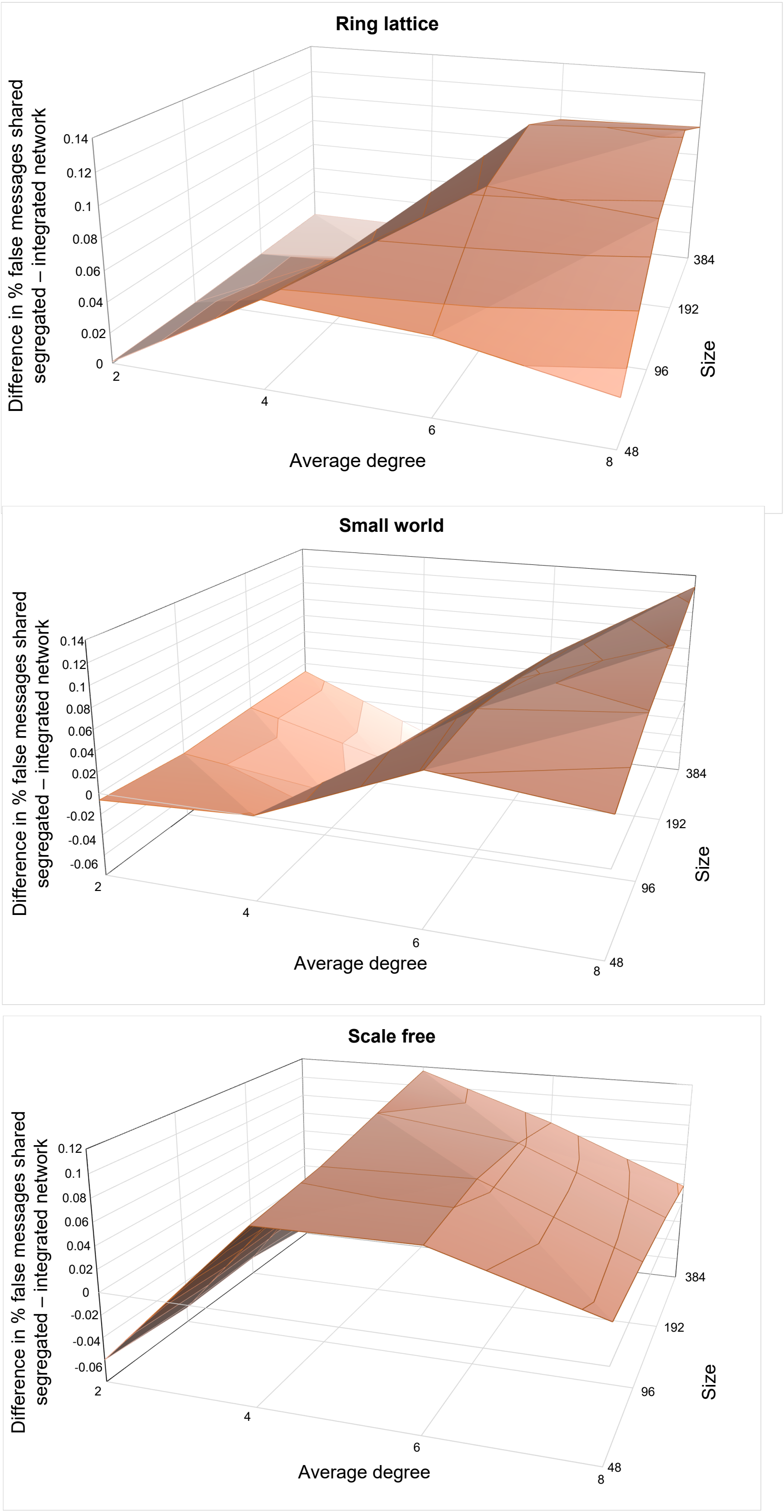

Fig. S6. Extrapolation to alternative network topologies. Variation of average degree and network size in ring lattice, small world ($r = 0.10$), and scale free networks ($\alpha = 1$).

Second, the segregation effect generalizes to other network typologies (Fig. S6). Network segregation increases the propagation of misinformation in networks with properties that allow some, but not all information to spread. When average degree is very high and network size small (as in the ring lattice with $N = 48$ and $k = 8$) path length is short and information transmits easily. In such instances, all information—even false messages with very low sharing probabilities—will circulate, regardless of segregation. In scale free networks with an average degree of 8, information transmission capabilities of the network are so vast that all information circulates and even the largest networks of $N = 384$ feature only minor segregation effects. At the other extreme, when average degree is low or networks are too large, no information will spread and, again, any segregation effect must be absent (as in lattice networks with $k = 2$). In small world networks with low degree and large size, the segregation effect can even reverse such that segregated networks facilitate the sharing of true over false information. This happens because these networks are so sparse that no information spreads unless it has a very high sharing probability and the network is segregated.

The segregation effect is positive and robust in each topology once average degree is adjusted in accordance with network size. This is because there are some high-plausibility messages that will spread reliably, irrespective of network segregation, and other messages with lower overall sharing probability that need clusters of susceptible individuals to diffuse. These results identify network topologies as particularly interesting, both theoretically and empirically, in which information with an intermediate level of virality has at least some chance to diffuse. Networks in which some, but not all information circulates are those that come closest to real-world networks and they are the ones where network segregation matters.

In additional simulations, we vary rewiring r in small world networks and the preferential attachment parameter α in scale free networks. Small world networks with $r = 0$ (i.e., ring networks without rewiring) feature high clustering and long path length, which hampers diffusion and only messages with high sharing probability p spread (Fig. S7). With increasing r, the network's information transmission capability increases and messages of low sharing probability diffuse in segregated networks. In scale free networks, the segregation effect decreases slightly in α. At extreme values of α, high-degree nodes become increasingly central—up to a point where maximum path length comprises only two or three links and all information must pass through the central nodes. Even here our finding is robust because, in segregated networks, low-p messages reach a central node more often, and the probability that the central node aligns with a message and shares it is higher. This result illustrates a case where seg-

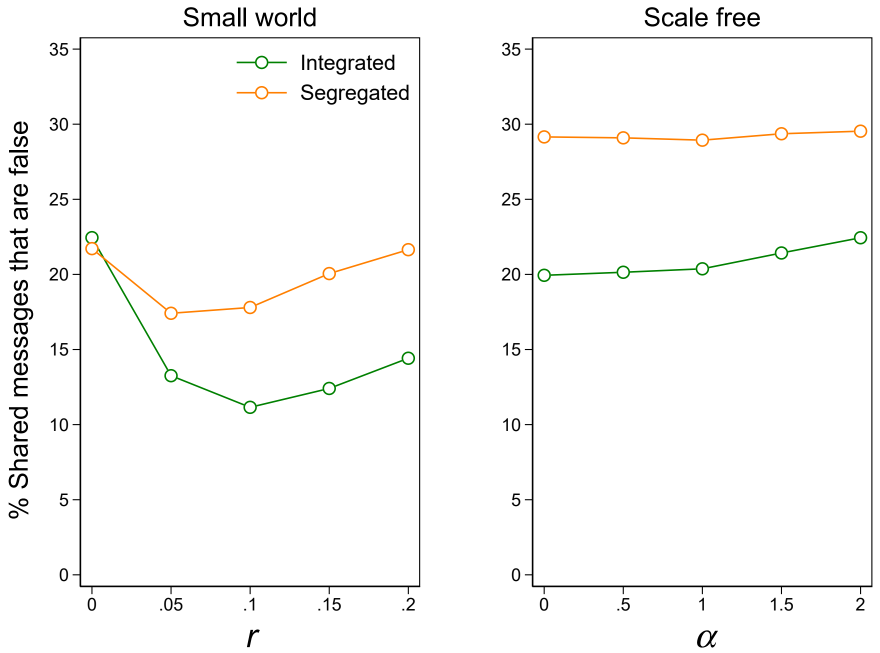

Fig. S7. Percentages of false sharing decisions in integrated and segregated simulated networks, as a function of rewiring r (small world network) and preferential attachment exponent α (scale free network). $N = 96$ and $k = 6$.

regation effects remain even when average path length is very short. When instead all nodes have a high degree, messages will diffuse regardless of segregation or messages' average sharing probability (see Fig. S6, bottom panel). But when the degree distribution is unequal, low-p messages only reach central nodes or will be shared by them if segregation is present. In conclusion, the segregation effect persists in networks with short path length, moderate average degree, and unequal degree distributions. Taken together, these results support generalizability of the segregation effect for the types of networks most commonly observed in the real world.

S4. Differences between liberals and conservatives

In additional analyses, we test for differences in attitudes and behavior between liberal and conservative participants, and we assess the degree to which each ideological camp is responsible for driving the reported experimental results. Note that the following results depend on our specific subjects' ideological affiliations, their partisan intensities and the content of our specific messages, neither of which was perfectly counterbalanced across ideologies, thus limiting inference.

Intensity of ideological self-identification. Upon entering the experiment, self-identified liberals on average reported a slightly stronger affiliation to their own camp (1.03) than conservatives did to theirs (0.87; t=3.36, p<0.001). These results are based on a $\{0, 1, 2\}$ recoding of the original 7-point scale in which we categorize participants as identifying "slightly" (0), "intermediately" (1), or "extremly" (2) with their camp. The two independent conditions elicit similar results (1.03 vs. 0.79).

Partisan sharing bias of misinformation. So far, we have computed ideological bias on the level of messages, as each message's difference in sharing rates among conservative and liberal subjects. Here, we compute ideological bias at the individual level as the difference in sharing rates of aligned and misaligned false messages for a given participant. We measure partisan misinformation sharing bias as the difference in sharing probabilities of aligned versus misaligned false messages, divided by two, in the non-incentivized independent condition where subjects rated all 16 false messages independently. On average, liberals exhibit higher levels of partisan misinformation sharing bias (0.16) than conservatives (0.08; t=3.90, p<0.001). While false conservative-leaning messages have higher bias than false liberal-leaning messages (averages from Table S1; liberal: $\bar{b}$ = 0.08; conservative: $\bar{b}$ = 0.15; t=3.63, p=0.003), liberal subjects are more inclined to prefer aligned misinformation over nonaligned misinformation than conservative subjects. This can be partly attributed to their higher level of ideological identification. The finding that aligned content, even if false, speaks to conservatives for its strong message bias falls in line with prior results from observational studies showing that conservative social media users are more likely to share content from partisan fake news sites[6,7]. But we also find that conservative subjects are more accepting of liberal-leaning content than liberal subjects are of conservative-leaning content. This finding adds to the debate about whether it is liberals or conservatives that engage more in news sharing across the ideological divide[8–10].

Which camp is driving the result? Fig. S8A shows the exposure of conservative and liberal nodes to aligning misinformation. In segregated networks, false messages spread well in their aligned clusters, both among conservative (77.6%) and liberal subjects (62.0%, yellow markers). In conservative clusters, aligned misinformation proliferates, despite low conservative subject bias, because of high message bias of conservative-leaning content. In liberal clusters, aligned misinformation spreads more, despite the low message bias of liberal-leaning content, because of the high partisan sharing bias among liberal subjects. Either combination adds to individual sharing probability, pushing messages of otherwise low sharing probability above the diffusion threshold.

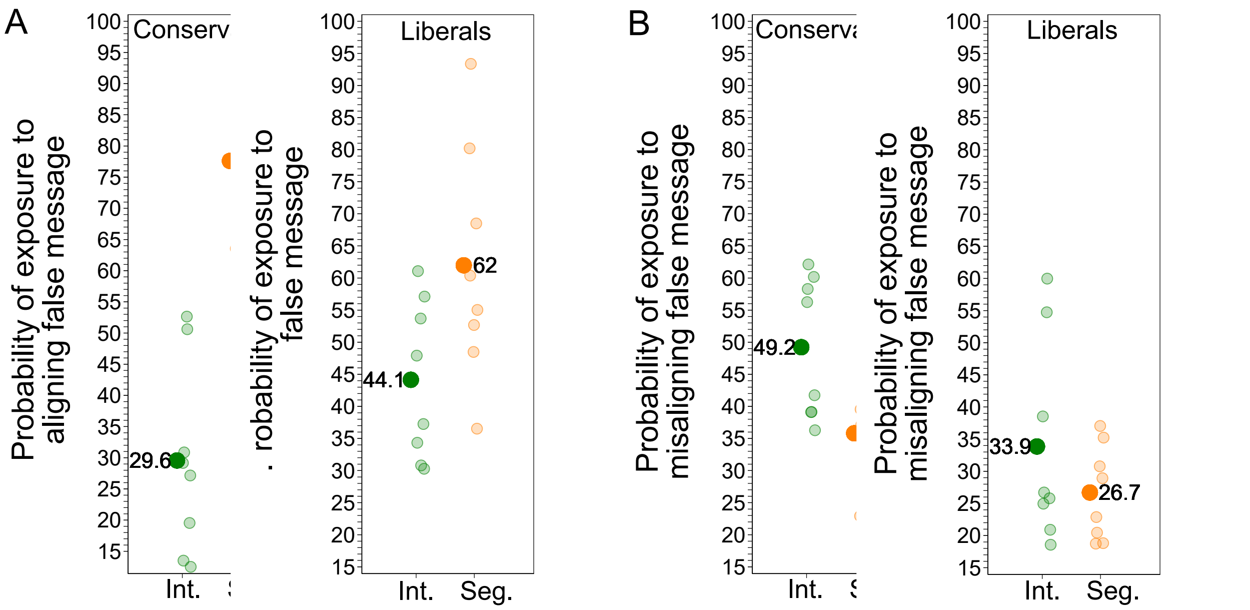

Fig. S8. Exposure to misinformation in integrated and segregated networks, differentiated by conservative and liberal participants. (*A*) Participants' individual probability of exposure to aligning false messages. (*B*) Exposure to misaligning false messages. Each shaded circle represents one network.

In integrated networks, most false conservative messages fail to spread (green markers in panels A, left (29.6%) and B, right (33.9%)) as a result of liberals' more pronounced partisan sharing bias. Liberals act as roadblocks as high message bias for conservative messages and high subject bias among liberals lead to very low sharing rates in this subpopulation. Liberal false messages, by contrast, diffuse reasonably well even in integrated networks. With lower message bias for false liberal-leaning content (0.08) and lower misinformation sharing bias among conservatives (0.08), conservative roadblocks are more lenient. In effect, there is more exposure to liberal misinformation in integrated networks than to conservative misinformation, and this holds among both liberal (44.1%) and conservative subjects (49.2%; green markers in panels A, right and B, left). Conservative clusters are somewhat permeable to liberal misinformation (35.8%, yellow marker in panel B, left) due to a combination of low conservative subject bias and low liberal message bias. Conservative misinformation, by contrast, diffuses much less in liberal clusters (26.7%, panel B, right). In sum, these nuanced results suggest that network segregation has more severe consequences for conservative false messages than for liberal ones, which is driven by unique features of both camps: liberals stop conservative misinformation in integrated networks, and conservative misinformation proliferates a lot more once segregation disperses liberal roadblocks. Conservatives, on the other hand, are too lenient with liberal misinformation, leading to liberal misinformation spread even when networks are integrated.

References

1. Chandler, J., Rosenzweig, C., Moss, A. J., Robinson, J. & Litman, L. Online panels in social science research: Expanding sampling methods beyond Mechanical Turk. *Behav. Res. Methods* **51,** 2022–2038 (2019).

2. American National Election Studies. *ANES Cumulative Data File Codebook 2019;* `https://electionstudies.org/data-center/`.

3. Centola, D. The spread of behavior in an online social network experiment. *Science* **329,** 1194–1197 (2010).

4. Watts, D. J. & Strogatz, S. H. Collective dynamics of 'small-world' networks. *Nature* **393,** 440–442 (1998).

5. Barabási, A.-L. & Albert, R. Emergence of scaling in random networks. *Science* **286,** 509–512 (1999).

6. Guess, A., Nagler, J. & Tucker, J. Less than you think: Prevalence and predictors of fake news dissemination on Facebook. *Sci. Adv.* **5,** eaau4586 (2019).

7. Grinberg, N., Joseph3, K., Friedland, L., Swire-Thompson, B. & Lazer, D. Fake news on Twitter during the 2016 U.S. presidential election. *Science* **6425,** 374–378 (2019).

8. Barberá, P., Jost, J. T., Nagler, J., Tucker, J. A. & Bonneau, R. Tweeting from left to right: Is online political communication more than an echo chamber? *Psychol. Sci.* **26,** 1531–1542 (2015).

9. Eady, G., Nagler, J., Guess, A., Zilinsky, J. & Tucker, J. A. How many people live in political bubbles on social media? Evidence from linked survey and Twitter data. *Sage Open* **9,** 2158244019832705 (2019).

10. Bail, C. A. *et al.* Exposure to opposing views on social media can increase political polarization. *Proc. Natl. Acad. Sci. U.S.A.* **115,** 9216–9221 (2018).
